# Genotype-Related Effect of Crowding Stress on Blood Pressure and Vascular Function in Young Female Rats

**DOI:** 10.1155/2014/413629

**Published:** 2014-03-05

**Authors:** Peter Slezak, Angelika Puzserova, Peter Balis, Natalia Sestakova, Miroslava Majzunova, Ima Dovinova, Michal Kluknavsky, Iveta Bernatova

**Affiliations:** Institute of Normal and Pathological Physiology, Centre of Excellence for Examination of Regulatory Role of Nitric Oxide in Civilization Diseases, Slovak Academy of Sciences, Sienkiewiczova 1, 813 71 Bratislava, Slovakia

## Abstract

This study investigated the influence of chronic crowding stress on nitric oxide (NO) production, vascular function and oxidative status in young Wistar-Kyoto (WKY), borderline hypertensive (BHR) and spontaneously hypertensive (SHR) female rats. Five-week old rats were exposed to crowding for two weeks. Crowding elevated plasma corticosterone (*P* < 0.05) and accelerated BP (*P* < 0.01 versus basal) only in BHR. NO production and superoxide concentration were significantly higher in the aortas of control BHR and SHR versus WKY. Total acetylcholine (ACh)-induced relaxation in the femoral artery was reduced in control SHR versus WKY and BHR, and stress did not affect it significantly in any genotype. The attenuation of ACh-induced relaxation in SHR versus WKY was associated with reduction of its NO-independent component. Crowding elevated NO production in all strains investigated but superoxide concentration was increased only in WKY, which resulted in reduced NO-dependent relaxation in WKY. In crowded BHR and SHR, superoxide concentration was either unchanged or reduced, respectively, but NO-dependent relaxation was unchanged in both BHR and SHR versus their respective control group. This study points to genotype-related differences in stress vulnerability in young female rats. The most pronounced negative influence of stress was observed in BHR despite preserved endothelial function.

## 1. Introduction

Stress is considered an etiological factor associated with the development of atherosclerosis and myocardial infarction as well as hypertension [1–3]. Research investigating mechanisms involved in stress-related cardiovascular disease and investigations concerning the roles of acute and chronic stress and their relation with the development of cardiovascular disease have attracted much attention [4]. However, despite this effort there are still ambiguous results concerning a causal association between stress and hypertension. Esler et al. [5, 6] concluded that mental stress is a cause of essential hypertension in humans; on the other hand, Sparrenberger et al. [7, 8] did not find psychosocial stress to be causally associated with hypertension. Discrepancies in results from human studies could arise from different stressors being considered, the intensity and duration of stress [9] and different populations investigated. Moreover, the age in which organism is exposed to aversive stimuli is another important factor. Regarding blood pressure (BP), many observations indicate that although the full manifestation of hypertension usually occurs later in life, its roots can be traced back to early ontogeny [10, 11]. One important period, common to both humans and rats, during which negative environmental factors affect later development of BP is the intrauterine period [11]. Additionally, data in animal studies indicate that the most important developmental window regarding BP after birth is between the sixth and eighth week of life in rats with a positive genetic predisposition to hypertension [12], that is, in the postweaning period, when rats have usually just been separated from the mother. The question, however, arises whether a given period of life might also be a critical window in subjects with a negative family history of hypertension or in those with a single hypertensive parent, and if stress acting at this age can accelerate an increase in their BP. As it is not possible to perform this kind of study in humans (i.e., to intentionally expose children to chronic stress and to control the duration and intensity of the stressor), we simulated such an experiment in rats using normotensive Wistar-Kyoto (WKY, both parents normotensive), spontaneously hypertensive (SHR, both parents hypertensive), and borderline hypertensive (BHR, mother hypertensive and father normotensive) rats. We used crowding as a stressor acting in the postweaning period. Although crowding is a relatively mild stressor, it has a considerable effect on the hypothalamic-pituitary-adrenal (HPA) axis and the sympathoadrenal system in adult rats [13–15]. However, in addition to stress-activated systems there are several anti-stress systems including the L-arginine/nitric oxide (NO) system [16]. It has been shown that stress-related hormones and mediators may affect both vascular NO production and degradation, and thus its bioavailability [17]. We have shown previously that chronic crowding stress increased NO production in the aortas of adult normotensive WKY males, which was associated with improved acetylcholine-induced relaxation [18]. Thus, the L-arginine/NO system supposedly protects normotensive rats from developing stress-induced hypertension. However, elevated NO production per se might not imply better NO bioavailability, especially under conditions of oxidative stress, which was observed in hypertensive rats [19] as well during acute or chronic stress [15, 20–23]. Additionally, it is of interest that despite significant sex-related differences in cardiovascular regulation [24], NO production [25], and stress-induced neuroendocrine and cardiovascular responses [26, 27], the majority of experimental studies have used male rats.

Thus, this study investigated the influence of crowding in postweaning WKY, BHR, and SHR female rats on the development of BP and NO-mediated vascular function. We hypothesize that exposure to crowding stress in the postweaning period could accelerate the development of hypertension in rats with a genetic predisposition to hypertension by altering oxidative status and/or NO production in the vascular system.

## 2. Material and Methods

### 2.1. Animals and Treatment

All rats, WKY, BHR, and SHR, were born in our certified animal facility in order to retain the same environmental background for all the rats. BHR were F1 offspring of SHR dams and WKY sires. After birth, the rats were kept together with their mother until the end of the fifth week of life. Then they were separated from the mother, divided according to sex, and females were randomly divided into the control and crowding-exposed group. Control rats were kept under standard conditions (more than 200 cm^2^/100 g of animal body weight) in groups of 4 rats per cage. Animals in the stress group were kept at five rats per cage and exposed to crowding stress, which was induced by reducing their living space to approximately 70 cm^2^ per 100 g of animal weight for two weeks. The precise size of the cages was adjusted using special cages with one flexible wall. This wall was moved to the appropriate position on the same days as the body weights of the rats were determined. All animals were maintained at an ambient temperature of 22 to 24°C under artificial light with a 12 h light/dark cycle during the whole experiment. The rats were fed a standard pellet food and had water and food *ad libitum*. The experiments were performed in accordance with European Community and NIH guidelines for the use of experimental animals and were approved by the State Veterinary and Food Administration of the Slovak Republic.

### 2.2. Blood Pressure and Heart Rate Measurements

Systolic blood pressure (BP) and heart rate (HR) were measured indirectly by tail-cuff plethysmography between 08.00 and 11.00 a.m. as was described in detail previously [15]. In order to minimize the influence of nonspecific stress on BP measurements, all rats were handled and accustomed to the tail-cuff procedure before experimentation. Each value was calculated as the average of five measurements. BP and HR values were measured at the beginning of the experiment (basal) and after the 1st and 2nd weeks of the experiment. Body weight (BW) was determined on the same days.

### 2.3. Plasma Corticosterone Measurements

Rats were sacrificed by decapitation after brief CO_2_ anesthesia between 07.30 and 09.30 a.m. [15]. Blood samples were collected in heparinized test tubes and immediately centrifuged at 850 g for 10 min at 4°C. Plasma samples were then stored at −80°C until analysis. Plasma corticosterone (pCort) was measured in duplicates using 20 *μ*L of plasma with an enzyme immunoassay kit (Arbor Assays, Ann Arbor, MI, USA), according to the manufacturer's instructions.

### 2.4. Vascular Reactivity Measurements

Femoral arteries were carefully dissected, immersed, and transferred to cold physiological salt solution (PSS) and then cleaned to remove the adipose and connective tissues [15]. Arterial segments (1.0 to 1.5 mm long) were mounted in a small vessel wire myograph (Dual Wire Myograph System 410A, DMT A/S, Aarhus, Denmark). Dissection, mounting, and normalization of each vessel were performed according to [28]. Femoral artery reactivity measurements were performed as described previously [15]. Briefly, the experimental protocol consisted of the following steps. (1) PSS in the myograph chamber was changed to KPSS (i.e., PSS in which NaCl was exchanged for an equimolar concentration of KCl 125 mmol/L) and contraction was measured. (2) Ten *μ*mol/L of norepinephrine (NE) was added and the contraction plateau value was recorded. (3) The artery was preconstricted with serotonin (1 *μ*mol/L). When the contraction of the artery was steady, increasing concentrations of the vasodilator acetylcholine (ACh, from 0.001 to 10 *μ*mol/L) were added in a cumulative manner and the endothelium-dependent relaxation response curve was recorded. (4) The same experiment was repeated after 25 min preincubation with the NO synthase inhibitor N^G^-nitro-L-arginine methyl ester (L-NAME, 300 *μ*mol/L) in the bath medium. (5) After serotonin preconstriction (1 *μ*mol/L), the NO donor sodium nitroprusside ([29], SNP, 0.001 to 10 *μ*mol/L) was added cumulatively and the relaxation response curve was recorded. (6) Finally, PSS was changed to KPSS and, after reaching the contraction plateau, NE (10 *μ*mol/L) was added and the maximal contraction of the artery was recorded. The artery was washed out four times with PSS and stabilized for 20 min after each step. All concentrations were expressed as final concentrations in the myograph chamber. All chemicals used were purchased from Sigma-Aldrich (Germany) and Merck Chemicals (Germany).

### 2.5. Nitric Oxide Synthase Activity

NO synthase (NOS) activity was determined by [^3^H]-L-arginine conversion to [^3^H]-L-citrulline (MP Biomedicals, USA) in crude tissue homogenates of aorta, as described previously [15] and was expressed as pmol/min/mg of protein.

### 2.6. Superoxide O_2_^−^ Measurement

The assay was performed as described previously [30] with some modifications. The aortic rings were cut (10–15 mg), cleansed of connective tissue, and placed into ice cold PSS. Lucigenin (50 *μ*mol/L) as well as tissue samples alone were added to PSS bubbled with pneumoxide (5% CO_2_ and 95% O_2_) at pH 7.4 and 37°C, and preincubated in the dark for 20 min. After preincubation, either background chemiluminescence or chemiluminescence produced by the aortic rings was measured for 6 min using a TriCarb 2910TR liquid scintillation analyzer (Perkin Elmer). Background counts were subtracted from values obtained from the samples. Results were expressed as counts per minute per mg of tissue (cpm/mg). All chemicals used were purchased from Sigma-Aldrich (Germany) and Merck Chemicals (Germany).

### 2.7. Statistical Analysis

Final body weight, heart rate and blood pressure, relative weight of the left ventricle and adrenal glands, normalized diameter, basal tension, and contraction data as well as the maximal response (*E*_max⁡_) and the concentration that produced a half maximal response (pD_2_) were analyzed using two-way ANOVA and Bonferroni post-hoc test. Differences between concentration response curves were assessed with two-way repeated measures ANOVA and vertical contrast using Bonferroni adjustment. The SNP-induced vasorelaxant data were fitted by four-parameter logistic function using GraphPad Prism 5.0 (San Diego, CA, USA). To determine the depression in relaxation at higher concentrations of ACh in the ACh dose-response curves, the maximal response to ACh and the responses at subsequent ACh concentrations were compared using Dunnett's test (Figure 3). Because of the inherent non-normality of pCort data, these were analyzed using a generalized linear model (Gamma distribution, logarithmic link function in R v.2.15.0, http://www.r-project.org/). All values (except for pCort) are presented as mean ± SEM. pCort data are presented as mean ±95% confidence interval. The significance level of all tests was set at 5% (*α* = 0.05).

## 3. Results

### 3.1. Basic Biometric, Cardiovascular, and Biochemical Parameters

There were significant differences in BW (Table 1) among the WKY, BHR, and SHR groups at the end of the experiment (*F*_2,71_ = 5.7; *P* = 0.005). The effect of age on BW was significant in all groups investigated (main effect of age *P* < 0.001). Stress reduced BW gain in general (*F*_1,71_ = 27.8, *P* < 0.001 main effect of stress); however, a significant stress-related reduction in BW gain was observed only in BHR and SHR versus the respective control (Table 1).

**Figure 1:**
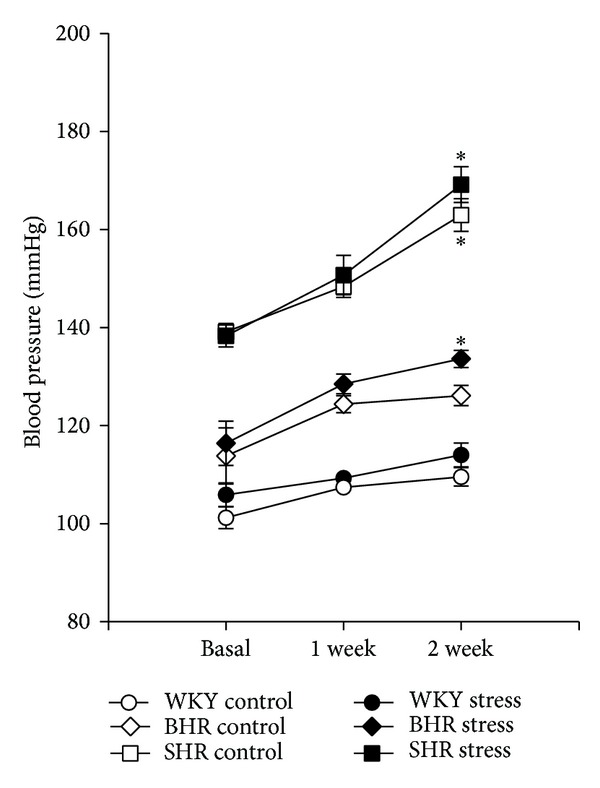
Effect of crowding stress on systolic blood pressure during two weeks of experiment. Data are presented as mean and SEM of 8–14 rats. *P < 0.05 versus the basal value. Differences among genotypes were significant (*F*_2,71_ = 29.0; *P* < 0.001).

Basal BP in 5-week WKY, BHR, and SHR rats was 103 ± 2, 115 ± 4, and 138 ± 2 mmHg, respectively, and there were significant differences among the genotypes (*F*_2,71_ = 29.0; *P* < 0.001). As the postweaning period was associated with body growth there were also significant age-related changes in BP (main effect of age *P* < 0.001). Although stress did not affect BP significantly, there was a consistent trend toward increased BP in all crowded rats compared to controls (Figure 1). However, there was a significant effect of time (*F*_2,71_ = 9.6; *P* < 0.001) and a time-strain interaction (*F*_4,71_ = 3.0; *P* = 0.023). This analysis revealed nonsignificant time-related changes in BP in both WKY groups. In SHR, a significant elevation in BP was observed in both groups as compared to basal values (*P* < 0.001). In BHR, stress accelerated BP increase as opposed to the prestress value (*P* = 0.034), while no significant increase was seen in the control BHR (Figure 1). Regarding HR, there were significant genotype-related differences (*F*_2,71_ = 13.2, *P* < 0.001, main effect of genotype); however, age and stress failed to affect HR in all genotypes investigated (Table 1).

Plasma corticosterone concentration was significantly different among genotypes (*P* = 0.014), with the highest values observed in SHR rats (Table 1). Stress significantly increased pCort (*P* = 0.027, main effect of stress). Although we observed higher levels of pCort in all WKY, BHR, and SHR stressed rats than in their respective control groups, the difference was statistically significant only in BHR. Additionally, significant genotype-related differences in relative adrenal gland weight (AG/BW) were observed (*F*_2,70_ = 50.0; *P* < 0.001). BHR and SHR, both the control and stress groups, had higher AG/BW ratios than the WKY groups but there was no significant effect of crowding (Table 1).

Under control conditions, relative left ventricular weights were significantly higher in SHR versus both WKY (*P* < 0.001) and BHR, respectively (*P* < 0.02), and no effect of crowding was observed (Table 1).

**Figure 2:**
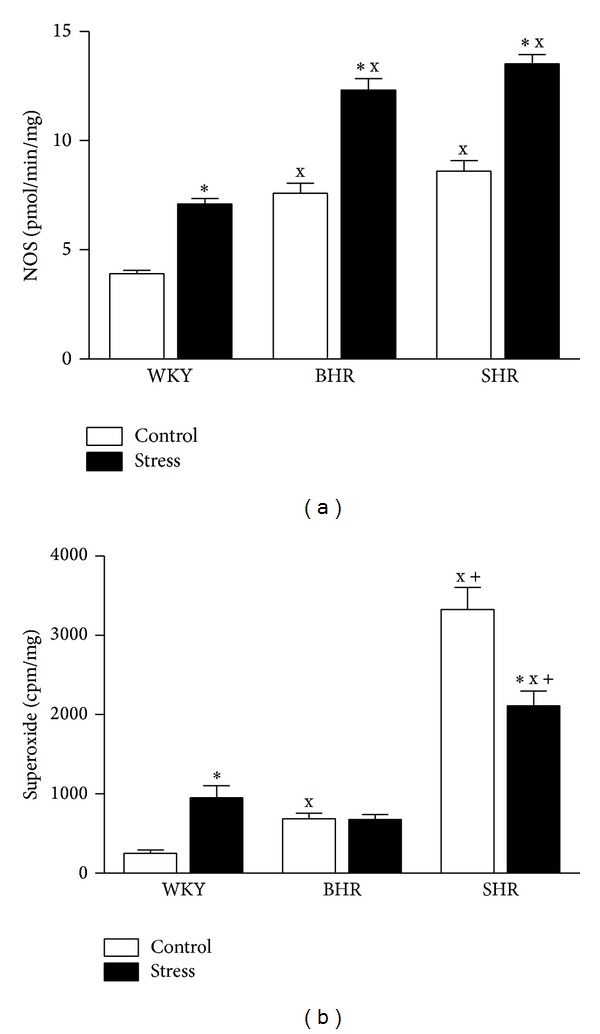
Effect of crowding stress on nitric oxide synthase (NOS) activity (a) and superoxide level (b) in the aortas of Wistar-Kyoto (WKY), borderline hypertensive (BHR), and spontaneously hypertensive (SHR) rats.^x^ *P* < 0.05 versus WKY respective group (control/stress); ^+^ *P* < 0.05 versus BHR respective group (control/stress); *P < 0.05 versus control group of the same genotype. Values represent mean ± SEM of six rats in each group.

Genotype and crowding had significant effects on NO synthase activity in the aorta: *F*_2,30_ = 18.2, *P* < 0.001, main effect of genotype; and *F*_1,30_ = 29.0, *P* < 0.001, main effect of stress. NOS activity was elevated significantly in both control BHR and SHR versus WKY, and stress increased NO production significantly in all genotypes investigated (Figure 2(a)).

Superoxide levels were also increased in control BHR and SHR versus WKY (*F*_2,30_ = 107; *P* < 0.001, main effect of genotype). In crowded BHR and SHR, superoxide levels were either unchanged (*P* = 0.72) or reduced (*P* < 0.001), respectively, versus the respective control group; however, in SHR they were still significantly higher versus stressed WKY and BHR (*P* < 0.05 versus both) (Figure 2(b)).

### 3.2. Vascular Function

**Figure 3:**
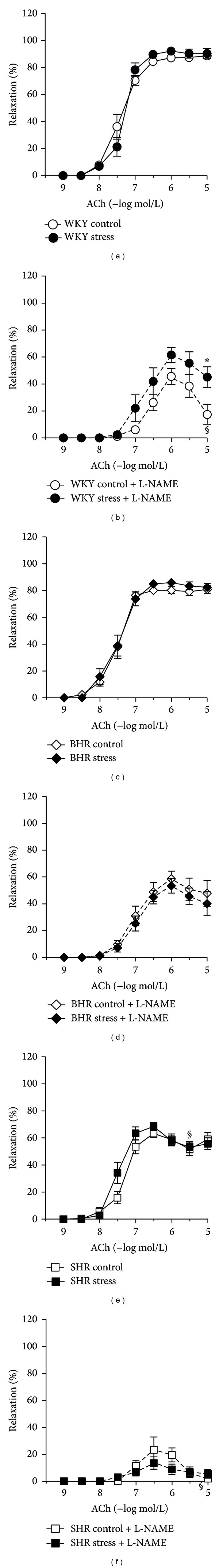
Effect of social stress on acetylcholine (ACh)-induced relaxation before ((a), (c), (e)) and after ((b), (d), (f)) pretreatment with the nitric oxide synthase inhibitor N^G^-nitro-L-arginine methyl ester (L-NAME) in the femoral arteries of young female Wistar-Kyoto (WKY), borderline hypertensive (BHR), and spontaneously hypertensive (SHR) rats. *P < 0.05 compared to the respective value in control rats;^§^ *P* < 0.05 compared to the maximal relaxation of the given group (indicating significant release of endothelium-derived contracting factors). Values represent mean ± SEM of 7–12 rats.

To investigate vascular function in the endothelium-intact femoral artery in individual genotypes as well as the effect of stress, we investigated normalized internal diameter and NE-, serotonin-, and KPSS-induced contractions as well as SNP-induced (Table 2) and ACh-induced relaxations (Table 2, Figures 3(a)–3(f)).

There were significant differences in the normalized diameter (ND) between BHR and SHR in both the control and stress groups, but crowding did not affect it significantly (Table 2). Norepinephrine and serotonin induced contractile responses in the femoral arteries. NE induced biphasic responses: a transient contraction (early response, phasic contraction) was followed by a depression in response almost to baseline value followed by a sustained contraction (delayed response, tonic contraction). There were differences among the rat strains in both phasic (*F*_2,48_ = 10.6; *P* < 0.001) and tonic (*F*_2,48_ = 9.3; *P* < 0.001) contractions, with significantly higher values observed in control BHR and SHR versus WKY. However, a significant effect of crowding was only observed in tonic contraction of BHR, which was attenuated versus control BHR (Table 2). On the other hand, no differences in serotonin-induced contractions were observed among the groups either before or after L-NAME pretreatment. The response of the arteries to serotonin after pretreatment with L-NAME was augmented compared to responses before L-NAME administration (main effect of L-NAME; *F*_1,92_ = 25.7; *P* < 0.001) and this effect was significant in both WKY groups as well as in the stressed BHR and SHR groups (Table 2).

Maximal KPSS-induced contraction (due to depolarization by K^+^) was not altered by crowding in any genotype versus its respective control group (Table 2).

Cumulative addition of exogenous NO donor SNP affected vasorelaxation similarly in all groups investigated. There were no differences in SNP-*E*_max⁡_ among the groups (Table 2). The sensitivity of the femoral arteries to SNP, determined as pD_2_, was significantly reduced in both SHR groups as compared to WKY and BHR (Table 2).

**Figure 4:**
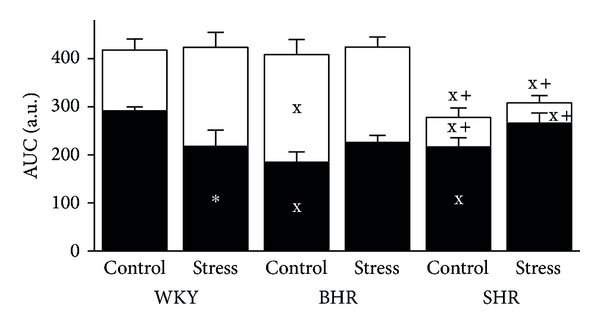
Effect of crowding stress on overall acetylcholine-induced relaxation and its NO-dependent (black bars) and NO-independent (white bars) components in the femoral arteries of Wistar-Kyoto (WKY), borderline hypertensive (BHR), and spontaneously hypertensive (SHR) rats. AUC—area under the curve; a.u.—arbitrary units; NO—nitric oxide; ^x^ *P* < 0.05 versus WKY respective group (control/stress); ^+^ *P* < 0.05 versus BHR respective group (control/stress); *P < 0.05 versus control group of the same genotype. Marks above the columns denote differences in overall relaxation and marks in the white (black) part denote differences in NO-independent (NO-dependent) relaxation, respectively. Values represent mean ± SEM of 7–12 rats.

There were significant genotype-related differences in overall ACh-induced relaxation expressed as either ACh-*E*_max⁡_ (Table 2, *F*_2,48_ = 116; *P* < 0.001) or the area under the ACh-induced dose-response curve (AUC) (Figure 4, *F*_2,48_ = 27; *P* < 0.001); significant reductions were observed in SHR versus both WKY and BHR (*P* < 0.001) for all comparisons. The main effect of stress on ACh-*E*_max⁡_ and AUC was not significant. The sensitivity of the femoral arteries to ACh, measured as ACh-pD_2_, was similar among the genotypes and stress failed to affect it compared to the control group of the same genotype. However, stress increased sensitivity to ACh in BHR and SHR versus stressed WKY (Table 2, *F*_2,48_ = 3.9; *P* < 0.03, effect of genotype-stress interaction).

Inhibition of NO synthase by L-NAME significantly reduced endothelium-dependent relaxations in all groups investigated (Figure 3). The NO-dependent component was reduced significantly in the young females with a genetic predisposition to high BP (*F*_2,48_ = 3.2; *P* < 0.05, main effect of genotype). On the other hand, the NO-independent component was significantly elevated in control BHR (*P* < 0.01) while a reduction was seen in control SHR versus WKY (*P* < 0.05; Figure 4). In addition, maximal relaxation after acute L-NAME pretreatment was significantly reduced in control SHR (27 ± 8%) versus WKY (47 ± 6%, *P* < 0.05).

There were significant differences in both the NO-dependent and NO-independent components of stressed WKY rats versus control (Figure 4). Crowding induced reduction of NO-dependent component of ACh-induced relaxation which was compensated by the elevation of NO-independent component in WKY, which was not observed in BHR and SHR (Figure 4).

## 4. Discussion

This study is the first to examine the effects of chronic stress on BP, superoxide, and NO production as well as vascular function in young normotensive, borderline hypertensive, and spontaneously hypertensive female rats in the postweaning period. Social stress produced by crowding, as described in this study, resulted in a generalized reduction in body weight gain as well as elevations in pCort and aortic NO production in all genotypes investigated. However, genotype-related differences in response to stress were observed in BP, superoxide concentration, and mechanism of vasorelaxation. Stress accelerated the increase in BP in BHR while no effect was observed in WKY and SHR. Vascular studies revealed overall endothelial dysfunction only in SHR, while normal endothelial function was seen in BHR. However, reduced NO-dependent relaxation was observed in both control BHR and SHR versus WKY. Interestingly, stress elevated superoxide concentration and reduced NO-dependent relaxation only in WKY, which was not observed in rats with elevated blood pressure.

This study was established to determine if stress acting in the postweaning period (i.e., in the period shortly after separation from the mother) can accelerate BP increases in rats. This developmental period was chosen as it is a critical developmental window in SHR rats [10]. Additionally, chronic noise, which can be considered a stressor, increased BP in 3-4 year-old children in kindergarten [31], that is, the period when children are usually separated from their mothers for the first time in their lives. The above-mentioned study suggested that exposure to stressful stimuli during the period of life when children are learning to be without maternal protection might negatively affect cardiovascular regulation. In addition, stress was shown to alter body weight, which might be expected to differ depending on the stressor, in humans [32, 33] and rats [34, 35]. In this experimental study, reduced age-related body mass gain and a consistent trend toward increasing BP was seen in all stressed groups. However, the most pronounced effect of stress was observed in rats with one hypertensive parent (mother in this case), in which pCort, body mass gain, and BP were all significantly affected. In SHR, the age-related increase in BP was similar in the control and stressed groups, despite reduced growth, suggesting that in this strain their genetic predisposition to hypertension is the dominant factor and stress does not alter BP under the given conditions.

This study showed that crowding is a mild but effective chronic stress model in postweaning female rats, similar to what we have shown previously in adult normotensive males [15, 18]. However, in this study we observed significant genotype-related differences in the activation of the HPA axis as determined by pCort concentration. In agreement with previously published studies, we observed a higher basal corticosterone concentration in young SHR animals compared to the WKY strain [36] but not in BHR [37]. Regarding the effect of stress, the main effect of crowding on pCort concentration was significant, but the most pronounced increase was observed in BHR, suggesting that young females of this genotype are particularly sensitive to this stressor. Insignificant increases in pCort in WKY and SHR rats (versus their respective control) at the end of the experiment can be explained by better adaptation, which protects organisms against detrimental effects of chronic stress. Adaptation to chronic stress has been observed in various stress models in humans as well as animals [38, 39], while an inability to adapt may result in diseased states. Similar to pCort, stress-induced elevation in BP was significant only in BHR. In agreement with this, other studies have shown higher pressor responses in adult BHR males to various stressors as compared to WKY [40–42]. Interestingly, differences in pressor adaptation to stress were shown to be related to the central effect of corticosterone. Bechtold et al. [41] found that endogenous corticosterone acts via hindbrain glucocorticoid receptors (GR) to enhance the pressor response to stress in adult BHR males but promotes adaptation in WKY. Moreover, prenatal dexamethasone treatment (a synthetic glucocorticoid analogue) increased baseline arterial pressure selectively in BHR in both sexes, but pCort increased only in female BHR [37]. With respect to our results, we assume that a similar corticosterone-mediated mechanism could be responsible for the differential effects of crowding on BP development in young WKY and BHR females. But to the best of our knowledge, no information is available in the literature on the role of hindbrain GRs in stress-exposed SHR.

In addition to the pressor effects, corticosterone is involved in modulating body mass. A study by Akana et al. [43] revealed the narrow range of pCort (10–75 ng/mL) that is compatible with a normal growth rate. In their study, reduced body weight gain was observed in young normotensive male rats when pCort exceeded this range. Indeed, pCort levels in the above-mentioned interval, together with normal body weight gain, were observed in stressed WKY and control BHR rats in this study, while elevated pCort concentrations in stressed BHR as well as in both SHR groups were associated with reductions in body mass gain. Crowding has also been reported to reduce body weight gain [44] via reduced food intake in both sexes [44, 45], which was also seen in young females in this study (data not shown).

Furthermore, there are studies showing that glucocorticoids may alter vascular function, and hypertension was shown to be the most significant negative side effect of chronic glucocorticoid treatment in humans [46, 47]. Additionally, inhibition of glucocorticoid release prevented acute mental stress-induced endothelial dysfunction [48]. Glucocorticoids were shown to downregulate NO production by limiting tetrahydrobiopterin (BH_4_) production [49] and eNOS gene transcription [50], which might result in endothelial dysfunction. Interestingly, in this experimental study, crowding did not affect maximal relaxation in any genotype, regardless of the pCort level, but it modified the mechanism of relaxation responses—its NO-dependent and independent components. The most pronounced alterations in the mechanism of vasorelaxation were observed in WKY, without alteration in BP, suggesting successful allostasis [51]. However, the absence of an influence of stress on overall relaxation in BHR (versus control), together with reduced NE-induced constriction, suggests that nonvascular mechanisms are involved in the acceleration of hypertension development seen in this genotype. In contrast, young control SHR females developed endothelial dysfunction, with reductions in both the NO-dependent and NO-independent components of relaxation (versus WKY), and stress failed to modify these changes. A finding of a reduced NO-independent component of ACh-induced relaxation in young SHR females is in agreement with our previous observation in adult SHR males [52]. In contrast, findings of a reduced NO-dependent component of ACh-induced relaxation in young control SHR and BHR females are in opposition with our recent observations of a positive correlation between BP and the NO-dependent component of relaxation in adult male BHR and SHR [52]. Whether this discrepancy results from the animals' different ages, sexes, or both remains to be elucidated in further studies, yet methodological aspects can be excluded [23] as the same methods were used in both our studies.

Oxidative stress, which seems to play an important role in the development of endothelial dysfunction [23], is another parameter that was shown to be modulated by glucocorticoids [50]. In this study, elevated superoxide concentration in the aorta was observed under control conditions in both genotypes with a predisposition to hypertension; however, it was more pronounced in SHR than in BHR. Regarding crowding stress, it elevated superoxide in WKY, in agreement with studies that found oxidative damage in normotensive rats exposed to chronic stress [22, 53, 54]. Interestingly, crowding stress had no effect in BHR and yet reduced superoxide concentration in SHR versus its respective control group. However, the level of superoxide in stressed SHR was still approximately two-fold higher compared to WKY and BHR under both control and stress conditions. The reason for this difference is unknown but we assume it may result from genotype-related differences in antioxidant defense systems. Indeed, we found elevations in plasma superoxide dismutase (SOD) activity and reduced lipoperoxide formation in blood as well as increased expression of SOD 1 and 2 in the kidneys of both BHR and SHR females exposed to stress, which was not observed in WKY (data not shown). However, neither elevated vascular NO synthase activity, in association with reduced superoxide level, was able to significantly improve NO-dependent relaxation in stressed SHR, which may be related to guanylate cyclase desensitization [55].

Regarding control SHR, despite enormous research, the exact mechanism of initiation of hypertension in this strain is still unclear. Our data suggest that elevated BP might be associated with alterations in corticosterone concentration or signaling and/or increased superoxide levels which might further lead to sympathetic activation and acceleration of the renin-angiotensin-aldosterone system [56, 57] and thus to an accentuated release of reactive oxygen species (ROS). Enormous ROS production can be implicated in the increased release of the endothelium-derived contracting factors, which may play a crucial role in the development of NO-independent endothelial dysfunction in genetic hypertension [58]. Moreover, possible structural changes in the vasculature of SHR and altered cell-to-cell communication may both participate in altered vascular function observed in SHR rats.

All together, these alterations may damage endothelial function, despite elevated vascular NO production, which would lead to increased peripheral resistance and the development of hypertension disease. If such is the case, how might the elevated BP in control BHR be explained when its basal corticosterone level was similar to that in WKY? It might be explained by elevated sensitivity to everyday stressors, which can be associated with transient periods of high corticosterone levels even under control conditions. As these periods were interrupted by periods of relief, the elevation of ROS could be relatively mild. Under such conditions the development of endothelial dysfunction would be delayed while the central effects of corticosterone acting via GRs might be responsible for the mild increase in BP observed in BHR. In addition, disruption or resetting of the baroreflex [42] may also participate in the gradual increase of BP in BHR. In such a situation, exposure of BHR to chronic social stress, associated with long-term HPA activation and imbalance in sympathovagal control [42], could accelerate the increase in BP as was observed in this study. The absence of signs of endothelial dysfunction in young BHR females exposed to crowding might also be explained by the relatively short duration and intensity of the stress used in this study, which may not be sufficient to exceed the adaptive mechanisms protecting vascular function, such as elevated NO production and reduced NE-constriction. Reduced NE-induced constriction in BHR may result from augmented sensitivity and number of *β*-2 adrenergic receptors due to elevated corticosterone [59] which may blunt *α*-1 adrenergic receptor-mediated constriction. Yet prolongation of stress could be associated with later development of NO-independent endothelial dysfunction, as we observed previously in WKY males exposed to 12 weeks of crowding [15]. Another possible explanation for the lack of stress-induced endothelial dysfunction is that the increasing estrogenic activity of young females at this age protects them from its development [60, 61].

## 5. Conclusions

In conclusion, this study resulted in several important findings. First of all, it points out that rats without a predisposition to hypertension were able to adapt to the given stressor via successful allostasis. Second, we showed significantly higher corticosterone and superoxide concentrations in young SHR females, which may play a role in the early development of endothelial dysfunction and hypertension in this strain. Furthermore, this study showed that the genetic predisposition to hypertension in SHR is the dominant factor in hypertension developing and stress did not aggravate the increase in BP under the given conditions. Additionally, we confirmed an elevation in vascular NO production after chronic stress exposure, which can act as an antistress mechanism in young females. However, the most important result of this study is that exposure to stress during a sensitive developmental period (in rats between the fifth and seventh weeks of life) can accelerate the inevitable increase in BP in juvenile females that are the offspring of hypertensive mothers and normotensive fathers. These females were more vulnerable to stress than female offspring of two normotensive or two hypertensive parents. Data suggested that their susceptibility to stress-induced hypertension resulted from the central effects of corticosterone rather than from altered vascular function.

On balance then, this study suggests caution in chronic exposure to stressors in childhood and juvenescence, especially in individuals where one parent suffers from hypertension. Although the progress of hypertension is usually slow and the disease is fully manifested in adulthood, it might originate from stressful environmental conditions in early periods of life.

## Figures and Tables

**Table 1:** Basic biometric and cardiovascular parameters and plasma corticosterone levels of young female Wistar-Kyoto (WKY), borderline hypertensive (BHR), and spontaneously hypertensive (SHR) rats exposed to crowding.

	WKY		BHR		SHR	
	Control	Stress	Control	Stress	Control	Stress
BW basal (g)	107 ± 4	105 ± 5	87 ± 3	96 ± 3	78 ± 2^x^	79 ± 3^x+^
BW final (g)	158 ± 4	154 ± 4	159 ± 3	146 ± 4*	121 ± 4^x+^	112 ± 3^x+^
BW gain (g)	51 ± 3	49 ± 4	73 ± 3^x^	50 ± 2*	43 ± 2^+^	33 ± 2^∗x+^
Final HR (bpm)	422 ± 16	418 ± 11	440 ± 25	463 ± 14	497 ± 17^x^	506 ± 31^x^
LVM/BW (mg/g)	1.93 ± 0.07	2.08 ± 0.10	1.93 ± 0.06	2.12 ± 0.07	2.51 ± 0.07^x+^	2.52 ± 0.07^x+^
AG/BW (mg/g)	0.24 ± 0.01	0.25 ± 0.01	0.28 ± 0.01^x^	0.28 ± 0.01^x^	0.29 ± 0.01^x^	0.29 ± 0.01^x^
^ 1^pCort (ng/mL)	68 ± 25	76 ± 22	58 ± 17	393 ± 331^x∗^	243 ± 47^x+^	287 ± 136^x^

Values represent mean ± SEM (except for pCort) of 8–14 rats. BW: body weight; HR: heart rate; LVM/BW: left ventricular mass-to-body weight ratio; AG/BW: adrenal gland-to-body weight ratio; pCort: plasma corticosterone; ^x^ *P* < 0.05 versus WKY respective group (control/stress); ^+^ *P* < 0.05 versus BHR respective group (control/stress); *P < 0.05 versus control group of the same genotype. ^1^Because of the inherent non-normality of pCort, these data were analyzed using a generalized linear model (Gamma distribution, logarithmic link function) and they are presented as mean ± 95% confidence interval.

**Table 2:** Basic vascular parameters, constrictions, and relaxations of the femoral artery in young female Wistar-Kyoto (WKY), borderline hypertensive (BHR), and spontaneously hypertensive (SHR) rats exposed to crowding.

	WKY		BHR		SHR	
	Control	Stress	Control	Stress	Control	Stress
ND (*µ*m)	579 ± 12	574 ± 15	630 ± 14	595 ± 10	537 ± 23^+^	538 ± 11^+^
Basal tension (kPa)	2.04 ± 0.21	1.92 ± 0.26	2.82 ± 0.20	2.24 ± 0.24	2.77 ± 0.53	2.84 ± 0.32
NE—phasic (kPa)	1.34 ± 0.27	2.42 ± 0.47	4.72 ± 0.64^x^	3.75 ± 0.53	5.01 ± 1.05^x^	5.75 ± 0.73^x+^
NE—tonic (kPa)	0.15 ± 0.04	0.35 ± 0.15	6.36 ± 1.78^x^	2.36 ± 0.63^x∗^	7.42 ± 2.02^x^	7.14 ± 1.74^x+^
Ser (kPa)	19.0 ± 0.6	19.9 ± 1.1	22.0 ± 1.5	23.7 ± 1.7	23.4 ± 1.7	21.9 ± 1.1
Ser—after L-NAME (kPa)	24.7 ± 0.5^†^	24.3 ± 1.7^†^	25.0 ± 1.8	28.9 ± 1.8^†^	27.7 ± 2.1	27.4 ± 1.3^†^
KPSS (kPa)	25.1 ± 1.2	25.0 ± 2.2	26.6 ± 2.7	32.6 ± 2.6^x^	34.2 ± 2.8^x+^	29.2 ± 2.2
SNP *E*_max⁡_ (%)	99 ± 1.1	96 ± 1.3	98 ± 1.5	97 ± 2.0	98.0 ± 2.0	95 ± 2.3
SNP pD_2_ (−log(mol/L))	7.96 ± 0.05	8.07 ± 0.06	8.09 ± 0.09	7.99 ± 0.11	7.69 ± 0.07^x+^	7.63 ± 0.07^x+^
ACh *E*_max⁡_ (%)	88 ± 1.9	91 ± 1.8	81 ± 1.9	85 ± 2.1	58 ± 1.9^x+^	60 ± 1.9^x+^
ACh pD_2_ (−log(mol/L))	7.39 ± 0.04	7.30 ± 0.03	7.50 ± 0.04	7.49 ± 0.05^x^	7.36 ± 0.05	7.53 ± 0.05^x^

Values represent mean ± SEM of 7–12 rats. ND: normalized diameter of the femoral artery at 13.3 kPa; NE: norepinephrine (10 *µ*mol/L); Ser: serotonin (1 *μ*mol/L); L-NAME: N^G^-nitro-L-arginine methyl ester; KPSS: PSS in which NaCl was exchanged for an equimolar concentration of KCl; SNP: sodium nitroprusside; ACh: acetylcholine; ^x^ *P* < 0.05 versus WKY respective group (control/stress); ^+^ *P* < 0.05 versus BHR respective group (control/stress); *P < 0.05 versus control group of the same genotype; ^†^ *P* < 0.05 versus the same group before L-NAME.
